# Angiogenesis in Paget's Disease of the Vulva and the Breast: Correlation with Microvessel Density

**DOI:** 10.1155/2012/651507

**Published:** 2012-03-26

**Authors:** Patricia E. Ellis, Allan B. MacLean, L. F. Wong Te Fong, Julie C. Crow, Christopher W. Perrett

**Affiliations:** ^1^Department of Obstetrics and Gynaecology, Royal Free and University College Medical School (Hampstead Campus), Royal Free Hospital, Rowland Hill Street, London NW3 2PF, UK; ^2^Department of Obstetrics and Gynaecology, Royal Surrey County Hospital, Guildford, Surrey GU2 7XX, UK; ^3^Department of Histopathology, Royal Free and University College Medical School (Hampstead Campus), Royal Free Hospital, Rowland Hill Street, London NW3 2PF, UK

## Abstract

Our understanding of the pathogenesis of Paget's disease of the vulva and the breast remains limited. Current evidence supports the fact that angiogenesis plays an important role in the pathogenesis of several diseases. Therefore, we sought to define its role, as correlated with microvessel density, in Paget's disease of the vulva and the breast. Microvessels were analysed using anti-von Willebrand factor antibody in 105 cases of Paget's disease of the vulva and the breast comprising 71 cases of Paget's disease of the vulva, including 8 cases with invasive disease, and 34 cases of Paget's disease of the breast. The latter included 12 cases with DCIS, 5 cases with both DCIS and invasive carcinoma, and 6 with carcinoma alone. Eleven cases had no underlying tumour identified. Increased microvessel density was demonstrated in Paget's disease of the breast with DCIS and with carcinoma alone compared to Paget's disease of the breast alone, *P* < 0.08 and *P* < 0.013, respectively. There were no significant differences in microvessel density in the vulval cases. Neovascularisation is an important process in the development of Paget's disease of the breast. Other biological and molecular processes are more involved in the pathogenesis of Paget's disease of the vulva.

## 1. Introduction

The pathogenesis of Paget's disease of the vulva (PDV) and Paget's disease of the breast (PDB) continues to be an enigma. Despite many theories that have been put forward on their origins and disease progression, the pathogenesis of these two diseases still remains unclear. PDV is an intraepithelial in situ carcinoma which accounts for approximately 1% of all vulval neoplasms [1]. PDB accounts for 0.5–4% of all breast cancers. They are both characterised by the presence of large, pale neoplastic (Paget) cells which are seen within the epidermis of the vulva and the nipple epithelium, respectively. In 10–30% of PDV cases, an invasive adenocarcinoma is present. This is in contrast to PDB where the general consensus is that almost all cases are associated with an in situ or invasive ductal carcinoma. This is based on the epidermotropic theory; Paget cells are ductal carcinoma cells that have migrated up from the underlying carcinoma to the nipple [2]. This theory, however, does not account for the cases of PDB that have no underlying carcinoma [3]. Toker cells have been described as precursor cells of both mammary and extramammary Paget's disease. These cells are found in the basal layer of the epidermis and are adjacent to the lactiferous ducts in the nipple [4]. They also occur as a normal constituent of genital skin in association with mammary like glands of the vulva [5]. The idea that Toker cells are precursors of mammary and extramammary Paget's disease is disputed by differences in immunoprofile and morphological appearance compared to Paget cells [6, 7]. The concept that Paget cells are in fact malignant keratinocytes, which has been transformed in situ, has been put forward as the transformation theory [8]. The transformation theory is favoured for the histogenesis for PDV and for those cases in PDB without an underlying carcinoma.

Angiogenesis is the formation of new capillary blood vessels from preexisting vasculature. It proceeds and sustains tissue growth and as such is an important component in tumour growth and metastasis. The exact timing of the point at which angiogenesis occurs, in the growth and progression of tumours, is known as the angiogenic switch [9]. The pathways controlling this switch to the angiogenic phenotype in tumours are dependent on a net effect of stimulators and inhibitors of angiogenesis [10–12]. This involves cell migration, matrix degradation by various growth factors, and the proliferation of the endothelial cells [13–15]. Stimulators of angiogenesis include vascular endothelial growth factor (VEGF), platelet-derived endothelial growth factor (PD-ECGF), and angiopoietin [16–18]. Thrombospondin-1, VEGF inhibitor, and angiostatin are well-known inhibitors of angiogenesis [19, 20]. In a previous study, we examined the expression of PD-ECGF/TP and VEGF in PDV and PDB. PD-ECGF/TP was expressed in 41% of Paget cells in PDV and 55% in PDB. There was no significant difference in PD-ECGF/TP expression in PDV and PDB with or without DCIS or invasive disease. VEGF was not expressed by Paget cells [21].

Microvessel density (MVD) is a measure of tumour angiogenesis. Increased MVD has been shown to be associated with disease progression and metastasis in several cancers, including vulval, breast, and prostate cancers [22–24]. A variety of endothelial cell markers have been used to identify microvessels for the purpose of counting. The most commonly used include factor-VIII-related antigen (F8RA)/von Willebrand factor (vWF), CD31/PECAM-1, and CD34. F8RA forms part of the vWF complex and plays a critical role in the process of haemostasis [25]. CD31 (PECAM-1), a platelet-endothelial cell adhesion molecule, is a transmembrane glycoprotein involved in cell adhesion [26], and CD34 is a surface glycoprotein expressed in endothelial cells in lymphoid tissue [27]. This current study extends our previous studies on Paget's disease of the vulva and the breast. The aim was to establish whether angiogenesis, as correlated with microvessel density, is different in PDV and PDB with or without an underlying tumour. The identification of an association between these diseases and angiogenesis would increase our understanding of the biological processes involved and would help us to move closer in unravelling the pathogenesis of PDV and PDB.

## 2. Materials and Methods

### 2.1. Tissue Specimens

Ethical approval was granted by the Royal Free Hospital NHS Trust. Seventy-one cases of PDV, including 8 cases associated with invasive disease, and 34 cases of PDB, which included 12 cases with DCIS alone, 5 cases with both DCIS and invasive carcinoma, 6 with an underlying invasive carcinoma, and eleven cases of PDB without a DCIS or an underlying carcinoma (PDB alone), were analysed for the expression of microvessels using anti-von Willebrand factor antibody. These cases were retrieved from the Histopathology Department at the Royal Free Hampstead NHS Trust and from collaborators as listed in the acknowledgements. The cases were diagnosed and treated between 1984 and 2000.

#### 2.1.1. Immunohistochemistry

Immunohistochemical staining was performed using the streptavidin-biotin-peroxidase technique. Briefly, sections were deparaffinised in xylene and rehydrated in different percentages of ethanol up to distilled water for 10 min. 3% hydrogen peroxide was placed on the sections to block endogenous peroxidase for 10 min. They were then placed in distilled water for 10 min at 37°C.

 Antigen retrieval was performed using 12.5 mg of proteinase (bacterial protease Type 24, Sigma) in 100 mL of phosphate buffered saline (PBS) at 37°C for 10 mins. The tissue sections were then incubated at room temperature with monoclonal anti-human vWF antibody for 1 hr (1 : 40 dilution; clone F8/86, Dako, Ely, Cambs, UK), followed by incubation with the secondary antibody (biotinylated rabbit anti-mouse immunoglobulin E0354, Dako), dilution 1 : 400 for 45 min. All sections were then incubated with streptavidin-biotin-horseradish peroxidase complex (Dako), diluted 1 : 200 in Tris-buffered saline for 30 min. Antibody binding was visualised with a solution containing the chromogen 3,3′-diaminobenzidine (Sigma-Aldrich, Poole, Dorset, UK) for 8–12 min and then terminated with tap water. The sections were counterstained with Mayer's haematoxylin (Merck, Lutterworth, Leics, UK), dehydrated in methanol, cleared in xylene, and mounted in DPX. Human placenta was used as positive control, and for negative control, vWF was replaced with PBS. In this study, we used vWF as the endothelial cell marker of choice because of its consistent staining and the fact that it was less likely to react with other tissue components, such as macrophages, compared to CD31 and CD34 endothelial cell markers.

### 2.2. Microvessel Density Assessment

A single countable microvessel was considered as a brown staining endothelial cell or endothelial cell cluster that was separate from adjacent microvessels, tumour cells, and other connective tissue elements. Large vessels with lumina greater than approximately seven red blood cells were excluded from the count [28]. Blood vessels were detected by a method similar to Bosari et al. [28]. Briefly, areas of highest neovascularisation, that is, containing the highest number of capillaries and small venules per area (hot spots) were found by scanning the whole tissue section at low power (×40 and ×100) using a light microscope. Five fields in each section with the highest number of hot spots were selected. The highest vessel density (HVD) of five fields at ×200 field (0.74 mm^2^ under the light microscope) and ×400 field (0.17 mm^2^ under the light microscope) was recorded, and the average vessel density (AVD) was also recorded in these five fields at ×200 and ×400. This was repeated using the HVD and AVD of three fields. The area of HVD and AVD using five fields did not differ significantly from the values obtained using three fields and therefore the analysis was performed using three fields. Individual MVD was made at both ×200 and ×400 magnification within each hot spot. The MVD is confined to an area within 500 *μ*m of dermal tissue just beneath the basement membrane of the epidermis and expressed as HVD/AVD per mm^2^.

Sections were stained on 3 separate occasions to ensure reproducibility. Results were analysed by three independent observers (PEE, LFWTF, and JCC). In all cases, there was <5% variation in results between sections and observers.

### 2.3. Statistical Analysis

 Statistical analysis was performed using the Mann-Whitney *U* test to compare MVD expression between invasive and noninvasive cases of PDV and cases of PDB with DCIS and invasive carcinoma. The SPSS v15 software was used to conduct the analysis. A *P* value of <0.05 was considered significant.

## 3. Results

**Figure 1:**
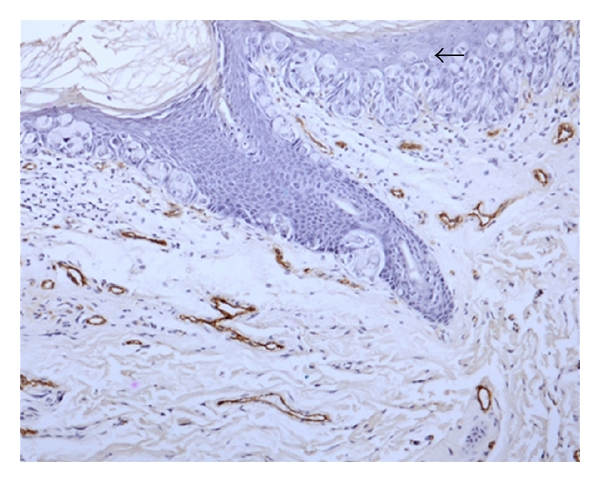
vWF expression demonstrating microvessels in PDB with DCIS (×200). Arrow : Paget cells.

**Figure 2:**
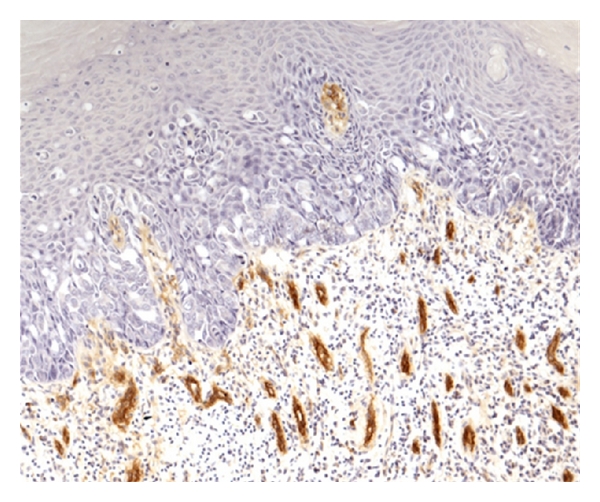
vWF expression demonstrating microvessels in PDV without invasive disease (×200).

**Figure 3:**
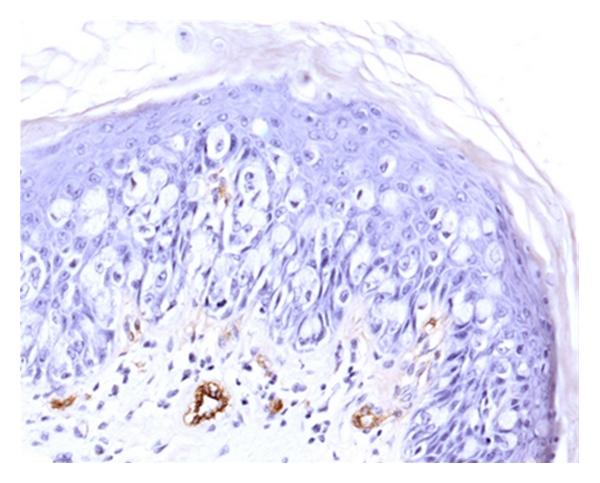
vWF expression demonstrating microvessels in PDV without invasive disease (×200).

The vascular endothelial cells were stained brown by the anti vWF antibody. Figures 1, 2, and 3 show the Paget cells and the surrounding stained microvessels in PDB and PDV. There were significant differences between PDB alone and PDB with DCIS, *P* < 0.008 at HVD  ×400 and *P* < 0.02 at AVD ×400; and between PDB alone and PDB with invasive cancer, *P* < 0.013 at HVD ×400 and *P* < 0.009 at AVD  ×400. The mean MVD ×400 was also higher in PDB with DCIS and invasive carcinoma compared to PDB alone but did not reach statistical significance. Similarly, the HVD and AVD ×200 magnification in cases of PDB with DCIS and PDB with invasive carcinoma were also higher compared to PDB alone. The mean HVD and AVD values in PDB are summarised in Table 1.

 The mean HVD at 200 and 400 magnification in PDV without invasive disease was 28.4 and 7.0, respectively, and 20.3 and 8.6 in PDV with invasive disease.  Table 2 demonstrates the mean values of the MVD in PDV. There appeared to be no significant difference in the MVD in intraepidermal PDV as compared with PDV associated with invasive disease.

## 4. Discussion

MVD has been used in several studies to investigate the role of angiogenesis in patients with cancer and has been shown to be a prognostic indicator for several tumours. The role of angiogenesis, as determined by MVD, has been examined in vulval lichen sclerosus, vulval intraepithelial neoplasia (VIN), and vulval cancer [29–31]. MVD was thought to be valuable prognostic marker for VIN 3 in determining progression to invasive disease [30]. Increased MVD was also associated with a poor prognosis in squamous cell carcinoma (SCC) of the vulva. It was not a useful parameter in determining potential malignant progression in vulval lichen sclerosus. In comparison, another study [32] did not demonstrate a positive correlation in vulval SCC with stage, survival, or pattern of invasion.

In breast cancer, there have also been conflicting results reported in the association with MVD and its role as a prognostic factor. It has been shown that intratumour MVD is an independent prognostic factor for breast carcinoma. The authors found a correlation between MVD and overall and relapse-free survival in patients with early-stage breast carcinoma [33]. Others have demonstrated the vessel density to be a significant prognostic indicator in node-negative and node-positive breast cancer [34]. A more recent study reported an increase in MVD between normal and benign hyperplastic breast tissue and between in situ and invasive carcinomas [35]. However, other studies have demonstrated a lower MVD in the breast carcinoma compared to the adjacent normal breast tissue [36] and were unable to find a relationship between MVD and breast metastases [37]. The inconsistent results reported in these studies may be due to the different techniques and endothelial cell markers used to measure tumour angiogenesis. A double-labelling technique was used to quantify MVD using CD34 or vWF [36]. vWF was used as the only endothelial cell marker [36] and a further study utilised CD31 as the endothelial cell marker and the Chalkley method to assess MVD [37]. The Chalkley method measures the relative area of vessel profile in a high-density region of the tumour as compared to MVD, which measures the density of the vessels [38]. Hollingsworth et al. [39] described a method using vascular volume to assess MVD. Determination of vessel density by vascular volume represents an average of the entire section rather than focusing on areas of most intense neovascularisation and therefore does not reflect the angiogenic activity of tumour cells or metastatic potential. To our knowledge, no other study has investigated MVD in PDV and PDB. Our findings suggest that neovascularisation is an important factor in the development of PDB but not in PDV. MVD as assessed by HVD and AVD ×400 magnification may be a useful parameter to determine which cases of PDB will have DCIS or invasive carcinoma disease present. Identifying those cases with a low MVD may allow a more conservative approach in the surgical management of PDB.

A different mechanism may be involved in the growth and progression of PDV. It is possible that in PDV, Paget cells can migrate and progress to invasive disease by utilising the existing vasculature, without the need for the formation of new blood vessels. This could explain why there was no difference in the microvessel counts between PDV with or without underlying invasive disease. Tumour progression in the absence of neoangiogenesis has been described by several authors [40–42]. “Cooption”, the utilisation of preexisting vasculature by tumours to obtain its blood supply and therefore to grow and progress, has been reported in malignant melanomas, brain metastases, and lung cancer. Döme et al. [40] demonstrated the incorporation of the existing host vascular plexus into a progressing malignant melanoma. Others have described growth of tumour cells in non small cell carcinoma of the lung without morphological evidence of neoangiogenesis [43] and the development of brain metastases, without the induction of sprouting angiogenesis, even in the presence of high levels of VEGF [41]. 

It is well documented that the growth and disease progression of many cancers is due in part to the loss of cell-cell adhesion. We have demonstrated that the cell adhesion molecule E-cadherin is significantly reduced (*P* = 0.039) in Paget's disease of the vulva cases with invasive disease when compared with Paget's disease of the vulva cases without invasive disease. E-cadherin expression was normal in PDB and there was no difference between those cases of PDB with or without DCIS or invasive disease [44]. These findings and the results from this current study demonstrate the critical steps involved in the pathogenesis of PDB and PDV may occur by different mechanisms.

In conclusion, this is the first study to assess MVD in PDV and PDB. MVD can be a useful parameter in determining the presence of PDB with or without DCIS or invasive disease. Additional work is needed to assess the relationship between MVD and other stimulators of angiogenesis in the pathogenesis of PDB and PDV. What impact the differences in the pathogenesis in PDB and PDV, as described in this study, have on the histogenesis of these two diseases remains to be clarified.

## Figures and Tables

**Table 1:** Mean values of the MVD in PDB.

Cases	*n*	HVD (×200)	AVD (×200)	HVD (×400)	AVD (×400)
PDB with DCIS	12	26.9 (13.7)	18.3 (12.3)	11.2 (3.1)	8.3 (3.1)
PDB with DCIS/invasive carcinoma	5	14.6 (10.0)	11.7 (8.9)	9.0 (4.6)	7.4 (3.8)
PDB with invasive carcinoma	6	20.8 (6.4)	15.3 (4.8)	17.2 (13.2)	14.3 (11.3)
PDB alone	11	19.1 (13.6)	13.9 (10.2)	7.2 (2.7)	5.1 (2.4)

*n*: number of cases. The SDs of the mean are given in brackets.

**Table 2:** Mean values of the MVD in PDV with and without invasive disease.

Cases	*n*	HVD (×200)	AVD (×200)	HVD (×400)	AVD (×400)
PDV without invasive disease	63	28.5 (16.4)	20.0 (14.04)	10.4 (5.9)	7.0 (3.8)
PDV with invasive disease	8	20.4 (19.5)	14.1 (13.3)	12.1 (9.4)	8.7 (5.4)

*n*: number of cases. The SDs of the mean are given in brackets.
